# Isolated Asymptomatic Short Sternum in a Healthy Young Girl

**DOI:** 10.1155/2014/761582

**Published:** 2014-07-20

**Authors:** Francesco Turturro, Cosma Calderaro, Antonello Montanaro, Luca Labianca, Giuseppe Argento, Andrea Ferretti

**Affiliations:** ^1^Orthopaedic Unit, S. Andrea Hospital, Sapienza University of Rome, Via di Grottarossa 1035, 00189 Rome, Italy; ^2^Radiology Unit, S. Andrea Hospital, Sapienza University of Rome, Via di Grottarossa 1035, 00189 Rome, Italy

## Abstract

Congenital sternal defects are rare deformities frequently associated with other anomalies of the chest wall and other organ systems. Although pectus excavatum, pectus carinatum, and cleft sternum can present as isolated deformity, in most cases they are associated with heart and inner organs anomalies and described as symptoms of syndromes like Marfan syndrome, Noonan syndrome, Poland anomaly, and Cantrell pentalogy. In contrast, the etiology of an isolated defect is not well understood. We observed a short sternum (dysmorphic manubrium, hypoplastic body, and complete absence of the xiphoid process) in a completely asymptomatic 13-year-old woman. A comprehensive instrumental exams panel was performed to exclude associated anomalies of the heart and of the other organ systems. The patient was completely asymptomatic and she did not need any medical or surgical treatment. To our knowledge, this is the first case of isolated short sternum reported in literature.

## 1. Introduction

Congenital sternal defects are rare deformities frequently associated with other anomalies of the chest wall and other inner organs. The etiology is not well understood [1].

We observed a type of isolated sternal defect to our knowledge not previously reported in literature, an abnormal development of the sternum in which a dysmorphic manubrium, a hypoplastic body, and the complete absence of the xiphoid process were identified, in an asymptomatic adolescent female without inner organs anomalies.

## 2. Case Presentation

**Figure 1:**
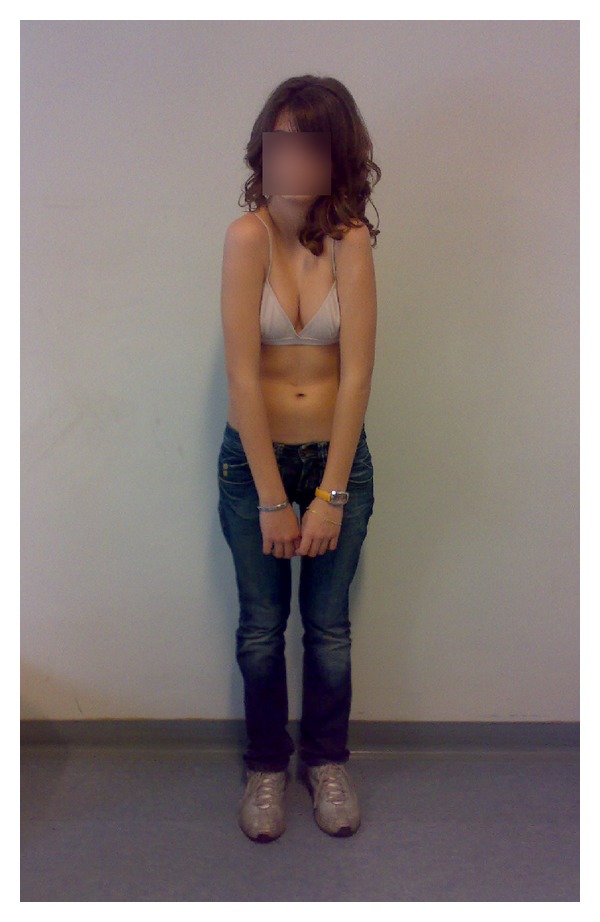
Clinical pictures of the patient.

**Figure 2:**
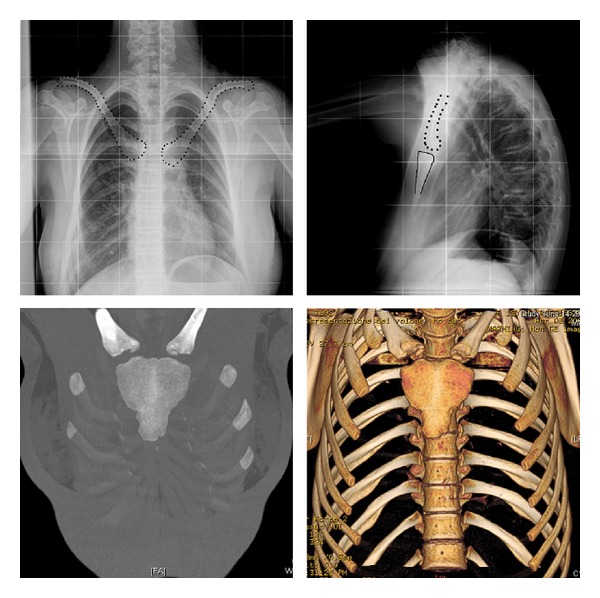
In the X-rays, the clavicles appear with a more vertical course (clavicles dotted line, sternum continuous line). The computed tomography scan with three-dimensional reconstruction documents the abnormal development of the manubrium, the presence of a hypoplastic body, and the absence of the xiphoid process. There is also an alteration of the sternoclavicular joint.

A healthy 13-year-old female was referred to our department for a postural kyphosis. The physical examination showed a healthy adolescent girl, 170 cm tall, with a normal and flexible kyphosis and no signs of spinal deformities. Only a hypermobility of the shoulder toward the anterior midline was observed (Figure 1). Because of the suspicion of a defect of the clavicles, a radiographic examination was performed. The images showed the presence of well-developed clavicles, but with anomalous oblique orientation and a short sternum (Figure 2). The subsequent chest and abdomen computed tomography scan showed the presence of abnormal manubrium, hypoplastic body of the sternum, and absence of the xiphoid process (Figure 2). Even more, the clavicles have taken abnormal morphological appearance with a more vertical pathway and the alteration of sternoclavicular joint was probably due to adaptation to the anatomy of the sternum. The intrathoracic and abdominal structures were reported to be normal. The echocardiography ruled out heart valve defect and large vessels anomalies.

The patient was completely asymptomatic and she did not need any medical or surgical treatment.

## 3. Discussion

Congenital abnormalities of the anterior thoracic wall comprise a spectrum of deformities such as thoracic ectopia cordis, cervical ectopia cordis, thoracoabdominal ectopia cordis, pectus excavatum, pectus carinatum, cleft sternum, and short sternum [1].

Although pectus excavatum, pectus carinatum, and cleft sternum can present as isolated deformity (less than 30 cases of isolated cleft sternum are reported in literature) [2–6], in most cases they are associated with heart and inner organs anomalies and described as symptoms of syndromes like Marfan syndrome, Noonan syndrome, Poland anomaly, and Cantrell pentalogy [1–7].

In contrast, the etiology of an isolated defect is not well understood. Many factors have been associated with murine models, like alcohol, riboflavin and methylcobalamin deficiency, and HOX4 gene disruption, but no significant associations have been reported in humans [2–7].

A case that is worth mentioning is that of Santa Rosa da Viterbo, in which a careful study of the well-preserved body showed that she was suffering from total agenesis of the sternum without other apparent malformations [8, 9].

We reported a case of an isolated short sternum in a healthy 13-year-old female patient. The clinical and instrumental examination ruled out other anomalies of inner organs related to known syndromes.

Although there is a large literature about the sternal defects and some reported cases of syndromic short sternum (trisomy 7, trisomy 18, trisomy 9, trisomy 12p, Cantrell pentalogy, Moebius syndrome, and Turner syndrome) [10–17], after a thorough review both in English and non-English languages, we did not find other cases of isolated short sternum; therefore, to our knowledge, this is the first case reported in literature.

## 4. Conclusion

Patients affected by chest wall deformity should be interdisciplinarily evaluated for other symptoms related to thoracic and abdomen organs to exclude eventual known syndromic causes.
